# Thanking our 2014 reviewers

**DOI:** 10.1186/s12935-015-0173-5

**Published:** 2015-03-05

**Authors:** Denys Wheatley

**Affiliations:** Director of BioMedES, Aberdeen, UK

## Abstract

The Editor-in- Chief would like to thank all the reviewers who contributed to the journal in 2014.

**Junaid Abdulghani**

United States of America

**Paul Agutter**

United Kingdom

**Joseph K Agyin**

United States of America

**Golo Ahlenstiel**

Australia

**Mohammed Aleskandarany**

United Kingdom

**Mikhail Alexeyev**

United States of America

**Holly Algood**

United States of America

**Sameh Ali**

Egypt

**Nicolas Andre**

France

**Ravindran Ankathil**

India

**Elif Damla Arisan**

Turkey

**Rukhsana Aslam**

Canada

**Kareem Azab**

United States of America

**Rodolphe Barrangou**

United States of America

**Giuseppina Barrera**

Italy

**Kevin Bennewith**

Canada

**Alfred Bere**

United States of America

**Thomas Binder**

Germany

**Yaroslav Blume**

Ukraine

**Lorella Bonaccorsi**

Italy

**Ricardo Borra**

Brazil

**Constance Brinckerhoff**

United States of America

**Barbora Brodska**

Czech Republic

**Elisa Callegari**

Italy

**Ana Paula Campanelli**

Brazil

**Ayse G Canseven**

Turkey

**Michele Caraglia**

Italy

**Silvia Carvalho**

Portugal

**Patrizia Casalini**

Italy

**Awalpreet Chadha**

United States of America

**Dimple Chakravarty**

United States of America

**Pithi Chanvorachote**

Thailand

**Emma Chapman**

United Kingdom

**Linjin Chen**

China

**Di Chen**

United States of America

**Stephen Chun**

United States of America

**Carlos J Ciudad**

Spain

**Ludovica Ciuffreda**

Italy

**Jean Clairambault**

France

**Ricardo Coletta**

Brazil

**David Conrad**

Canada

**Leah Cook**

United States of America

**Ricardo Correa**

United States of America

**Daniela Elena Costea**

Norway

**Alysha Croker**

Canada

**Cem Dane**

Turkey

**Karen Dane**

United States of America

**Jayasri Das Sarma**

India

**Jharna Datta**

United States of America

**Lester Davids**

South Africa

**Maryna de Kock**

South Africa

**Paul de Souza**

Australia

**Prasenjit Dey**

United States of America

**Isabelle Dhennin-Duthille**

France

**Violante Di Donato**

Italy

**Xiaoyan Ding**

China

**Rosa Divella**

Italy

**Nicole Doudican**

United States of America

**Yiannis Drosos**

United States of America

**Chandradhar Dwivedi**

United States of America

**Marzieh Ebrahimi**

Iran

**Martins Ekor**

Ghana

**Steve Elder**

United States of America

**Anat Erdreich-Epstein**

United States of America

**Jekaterina Erenpreisa**

Latvia

**Shinichi Esaki**

United States of America

**Fei Fei**

United States of America

**Zhou Fei**

China

**Zvi Fishelson**

Israel

**Marco Folini**

Italy

**Dimitrios Fotiadis**

Greece

**Christian Freise**

Germany

**Kai Fu**

United States of America

**Ota Fuchs**

Czech Republic

**Teruhiko Fujii**

Japan

**Mariateresa Fulciniti**

United States of America

**Mayuko Furuta**

Japan

**Koraljka Gall Troselj**

Croatia

**Michael Gallagher**

Ireland

**Elena Gallardo Martín**

Spain

**Rosaria Gangemi**

Italy

**Chenxi Gao**

United States of America

**Yong Gao**

China

**Manoj Garg**

Singapore

**Maria Gazouli**

Greece

**Paola Gazzaniga**

Italy

**Pengfei Ge**

China

**R Christopher Geiger**

United States of America

**Efstathia Giannopoulou**

Greece

**Veronique Giroux**

United States of America

**Nicola Giuliani**

Italy

**Munirathinam Gnanasekar**

United States of America

**Wolfgang H Goldmann**

Germany

**Susumu Goyama**

United States of America

**Thomas Greither**

Germany

**Kun Guo**

China

**Yige Guo**

United States of America

**Kwon-Soo Ha**

Korea, South

**Christina Hackl**

Germany

**Judith Hagenbuchner**

Austria

**Joerg Haier**

Germany

**Mu Hao**

China

**David Hein**

United States of America

**Toru Hiraga**

Japan

**Yi-Chiang hsu**

Taiwan

**Pavla Hublarova**

Czech Republic

**Douglas Hurst**

United States of America

**Sudhir Isharwal**

United States of America

**Tom Jackson**

United Kingdom

**Ruy Jaeger**

Brazil

**Morana Jaganjac**

Qatar

**John Jakupciak**

United States of America

**Tatiana Jazedje**

Brazil

**Yongfang Jiang**

China

**Xiaohua Jiang**

Hong Kong

**Jonathan Jones**

United States of America

**Shin-ichiro Kagami**

Japan

**Kazuharu Kai**

United States of America

**Galatea Kallergi**

Greece

**Jyotshna Kanungo**

United States of America

**Yasumasa Kato**

Japan

**Evan Keller**

United States of America

**Sofia Khaitlina**

Russian Federation

**Tobias Kiesslich**

Austria

**Thomas Kietzmann**

Finland

**Hark Kim**

Korea, South

**Yoji Kishi**

Japan

**Robert Kiss**

Canada

**Jorg Kleeff**

Germany

**Vera Kloten**

Germany

**Lars Oliver Klotz**

Germany

**Burkhard Kneitz**

Germany

**Junya Kobayashi**

Japan

**Takashi Kohno**

Japan

**Viswa Kalyan Kolli**

India

**Ling-Min Kong**

China

**Maritha Kotze**

South Africa

**Arpad Kovacs**

Hungary

**Muthusamy Kunnimalaiyaan**

United States of America

**Ajaikumar Kunnumakkara**

United States of America

**Leoni Kunz-Schughart**

Germany

**Elena Kurenova**

United States of America

**Toshihiro Kushibiki**

Japan

**Hiroyuki Kuwano**

Japan

**Ashakumary Lakshmikuttyamma**

United States of America

**Incilay Lay**

Turkey

**Fuyang Li**

United States of America

**Xiaoming Li**

China

**Evi Lianidou**

Greece

**Cheng-gong Liao**

China

**Delong Liu**

United States of America

**Yinkun Liu**

China

**Kaishan Liu**

China

**Yinkun Liu**

China

**Longshan Liu**

China

**Lawrence Loeb**

United States of America

**Emil Lou**

United States of America

**Shixin Lu**

China

**Hui-he Lu**

China

**Ochiba Lukandu**

Kenya

**Isabel Luthy**

Argentina

**Yoshihiko Maehara**

Japan

**Zaynah Maherally**

United Kingdom

**Ilaria Malanchi**

United Kingdom

**Gabriella Marucci**

Italy

**Masato Matsuoka**

Japan

**Loredana Mauro**

Italy

**James A McCaul**

United Kingdom

**Rheem Medh**

United States of America

**Parvin Mehdipour**

Iran

**Andreas Merdes**

France

**Bonhong Min**

Korea, South

**Toshinari Minamoto**

Japan

**Kiyoshi Misawa**

Japan

**Mitsuru Miyachi**

Japan

**Petr Mlejnek**

Czech Republic

**Zengnan Mo**

China

**David Mole**

United Kingdom

**Eiichi Morii**

Japan

**Taiki Moriyama**

Japan

**Dezhi Mu**

China

**Ping Mu**

United States of America

**Francis Mussai**

United States Minor Outlying Islands

**Shamaladevi Nagarajarao**

United States of America

**Rakesh Naidu**

Malaysia

**Shoji Natsugoe**

Japan

**Tamara Nikuseva-Martic**

Croatia

**Hongxiu Ning**

United States of America

**Makoto Noda**

Japan

**Joshua Obar**

United States of America

**Norma O'Donovan**

Ireland

**David Ornelles**

United States of America

**Iwata Ozaki**

Japan

**Xiufeng pang**

China

**Vassilis Papanikolaou**

Greece

**Palak Parekh**

United States of America

**Nicholas Pavlidis**

Greece

**Nives Pecina-Slaus**

Croatia

**Jun Peng**

China

**Lirong Peng**

United States of America

**Qiliu Peng**

China

**Guy Perkins**

United States of America

**Christian Petzelt**

Portugal

**Ulrich Pfeffer**

Italy

**Mark Pickard**

United Kingdom

**Smitha Pillai**

United States of America

**Pawinee Piyachaturawat**

Thailand

**Stefania Pizzimenti**

Italy

**Dusan Popadic**

Serbia

**Guy Pratt**

United Kingdom

**Vinesh kumar Puliyappadamba**

United States of America

**Wenbin Qian**

China

**Yi Qiu**

United States of America

**Xiong-Zhi Quan**

China

**Komal Ramani**

United States of America

**Stephan Reshkin**

Italy

**Carmela Ricciardelli**

Australia

**Alan Richardson**

United Kingdom

**Nicole Riddle**

United States of America

**Christoph Ritter**

Germany

**Maria Rizzo**

Italy

**Alexander Rölle**

Germany

**Achmad Chusnu Romdhoni**

Indonesia

**Aditi Roy**

India

**Ye-Chun Ruan**

Hong Kong

**Clinton Rubin**

United States of America

**Hannes Sallmon**

Germany

**Dario Sangiolo**

Italy

**Ronit Satchi-Fainaro**

Israel

**Michael Schuliga**

Australia

**Udo Schumacher**

Germany

**Ljiljana Serman**

Croatia

**Li Shen**

United States of America

**Gajanan Sherbet**

United Kingdom

**Gwo Tarng Sheu**

Taiwan

**Hidenori Shiraha**

Japan

**Chengchao Shou**

China

**Viji Shridhar**

United States of America

**Andriy Sibirny**

Ukraine

**Stephen Sims**

Canada

**Ajay Vikram Singh**

United States of America

**Ronit Sionov**

Israel

**Ondrej Slaby**

Czech Republic

**Carlos Sonnenschein**

United States of America

**Oleh Stasyk**

Ukraine

**Philipp Stawowy**

Germany

**Peter Steinberger**

Austria

**Rostyslav Stoika**

Ukraine

**Sangeetha Sukumari Ramesh**

United States of America

**Tuangporn Suthiphongchai**

Thailand

**Larry Suva**

United States of America

**Umesalma Syed**

India

**Cezary Szczylik**

Poland

**Bela Szende**

Hungary

**He Taiping**

China

**Akimitsu Takagi**

Japan

**Hiroshi Takemori**

Japan

**Leandro Thiago**

Brazil

**Erik (Rik) Thompson**

Australia

**Mangesh Thorat**

United Kingdom

**Qiangsong Tong**

China

**Meng-Feng Tsai**

Taiwan

**Po-lin Tseng**

Taiwan

**Mitchell Turker**

United States of America

**Kin Pong U**

Hong Kong

**Ruchan Uslu**

Turkey

**Patricia Valerio**

Brazil

**Tom Vanden Berghe**

Belgium

**Daniele Vergara**

Italy

**Farhad Vesuna**

United States of America

**Jeppe Vinther**

Denmark

**Tomislav Vladusic**

Croatia

**André O von Bueren**

Germany

**Nathan Wall**

United States of America

**Shanshan Wan**

United States of America

**Changxi Wang**

China

**Yu-li Wang**

United States of America

**Karl Welte**

Germany

**Zhi-Hong Wen**

Taiwan

**Zhenke Wen**

China

**Mike-Andrew Westhoff**

Germany

**Sally Wheatley**

United Kingdom

**Theresa Whiteside**

United States of America

**Sopit Wongkham**

Thailand

**William Wright**

United States of America

**Yi Wu**

China

**Zhe Bao Wu**

China

**Ning-shao Xia**

China

**Hongping Xia**

China

**Guoan Xiang**

China

**Guozhi Xiao**

United States of America

**Jinliang Xing**

China

**Lin Xu**

China

**Nobuo Yaegashi**

Japan

**Naoko Yamada**

Japan

**Munekazu Yamakuchi**

Japan

**Ju Yang**

China

**Jin-Ming Yang**

United States of America

**Xiaobo Yang**

China

**Zhi-Zhang Yang**

United States of America

**Gong Yang**

China

**Lin Ye**

United Kingdom

**Samantha Yeligar**

United States of America

**Venkat Laxmi Yeruva**

United States of America

**Ken Young**

United States of America

**Luyang Yu**

China

**Dahai Yu**

China

**Wei-dong Zang**

China

**Mariastella Zannini**

Italy

**Neven Zarkovic**

Croatia

**Maria Chiara Zatelli**

Italy

**Ingeborg Zehbe**

Canada

**Zihua Zeng**

United States of America

**Bo Zhang**

China

**Jiwang Zhang**

United States of America

**Kejun Zhang**

China

**Sen Zhang**

China

**Shi-Meng Zhang**

China

**Yingshe Zhao**

United States of America

**Shusen Zheng**

China

**Binhua Zhou**

United States of America

**Ying Zhu**

United States of America

**Ceniz Zihni**

United Kingdom

